# Predictive models of poly(ethylene-terephthalate) film degradation under multi-factor accelerated weathering exposures

**DOI:** 10.1371/journal.pone.0177614

**Published:** 2017-05-12

**Authors:** Abdulkerim Gok, David K. Ngendahimana, Cara L. Fagerholm, Roger H. French, Jiayang Sun, Laura S. Bruckman

**Affiliations:** 1 Department of Materials Science and Engineering, Gebze Technical University, Gebze, Kocaeli, Turkey; 2 Solar Durability and Lifetime Extension (SDLE) Research Center, Department of Materials Science and Engineering, Case Western Reserve University, Cleveland, Ohio, United States of America; 3 Center for Statistical Research, Computing & Collaboration (SR2c), Department of Epidemiology and Biostatistics, Case Western Reserve University, Cleveland, Ohio, United States of America; Duke University Marine Laboratory, UNITED STATES

## Abstract

Accelerated weathering exposures were performed on poly(ethylene-terephthalate) (PET) films. Longitudinal multi-level predictive models as a function of PET grades and exposure types were developed for the change in yellowness index (YI) and haze (%). Exposures with similar change in YI were modeled using a linear fixed-effects modeling approach. Due to the complex nature of haze formation, measurement uncertainty, and the differences in the samples’ responses, the change in haze (%) depended on individual samples’ responses and a linear mixed-effects modeling approach was used. When compared to fixed-effects models, the addition of random effects in the haze formation models significantly increased the variance explained. For both modeling approaches, diagnostic plots confirmed independence and homogeneity with normally distributed residual errors. Predictive R^2^ values for true prediction error and predictive power of the models demonstrated that the models were not subject to over-fitting. These models enable prediction under pre-defined exposure conditions for a given exposure time (or photo-dosage in case of UV light exposure). PET degradation under cyclic exposures combining UV light and condensing humidity is caused by photolytic and hydrolytic mechanisms causing yellowing and haze formation. Quantitative knowledge of these degradation pathways enable cross-correlation of these lab-based exposures with real-world conditions for service life prediction.

## Introduction

The reliability of photovoltaic (PV) modules is of critical importance [1] to the growing PV industry. The PV module polymeric backsheets play a critical role in power production, electrical safety, and lifetime performance [2, 3]. Today’s PV modules typically have a 25 year [4] product warranty based on pass/fail type standardized tests not designed for lifetime qualification. Environmental stressors present in all climatic zones, such as irradiance, heat, and humidity, drive degradation of polymeric components in PV modules and contribute to performance loss. One important degradation pathway of poly(ethylene-terephthalate) (PET) containing backsheets leads to PET embrittlement and cracking [5, 6] in addition to backsheet delamination [7, 8]. Degradation modes lead to loss of wet insulation resistance and loss of PV module integrity and contribute to other degradation and power-loss modes.

PET is highly susceptible to moisture and ultraviolet (UV) irradiance [9]. PET degradation mainly occurs via photolytic and hydrolytic cleavage of an ester bond resulting in decreased molecular weight with concomitant changes in crystallinity, morphology, discoloration (yellowing) and/or haze formation. Photodegradation and/or photo-oxidation mainly occurs via Norrish type I or Norrish type II reactions [10–13]. When kinetically controlled, such as autocatalysis due to active carboxyl end groups, hydrolysis becomes more complex [14–17]. Stabilizing additives attempt to reduce the degradation rate and increase the service lifetime. These additives are also subject to degradation and can introduce subsequent degradation pathways [18, 19]. A comprehensive understanding of degradation pathways of PET grades under multi-factor exposures is required.

Accelerated weathering exposures can provide useful information in shorter time periods, but the stressors (irradiance, heat, humidity) and their intensity levels must be chosen to avoid activation of unrealistic degradation modes and failure mechanisms not seen under real-world conditions. Lab-based accelerated tests typically study the effect of a well-controlled single stressor on previously defined degradation mechanisms and examine a single response. The standardized weathering exposures are viewed as an indicator of lifetime performance even though they were developed solely to identify defects arising from manufacturing processes. These tests lack quantitative information about degradation mechanisms, failures, or insights into how modules and/or materials will behave in outdoor service. We focus on identifying multiple degradation modes that arise under multi-factor exposure conditions using statistically informed study protocols to produce datasets of step-wise observational variables to build physical and statistical models [20, 21]. This approach is akin to machine learning methods that are being developed [22], to encompass and capture the temporal evolution and multiple mesoscale interactions associated with degradation over lifetime. These degradation science network models of mechanisms and pathways utilize a < Stress|Response > perspective [23, 24] and can provide a predictive framework for service life prediction [25–28].

Several predictive models have been discussed in the PV and material literature. These span from least squares analysis that produces empirical relationships for dielectric, mechanical, and chemical properties [29, 30], to multiple regression modeling of thermo-oxidative and outdoor weathering of polymers with diagnostic confirmation [31]. Hossain et al. [32] developed a multiple regression predictive model for the operating temperature of PV module microinverters with rank-ordered dependencies on ambient temperature and PV module temperature which is strongly affected by the solar irradiance. The time-temperature superposition principle was applied to degradation rates of PET and EVA (ethylene-vinyl acetate) polymers under accelerated weathering exposures [33], but accurate determination of activation energies is a challenge. Köhl et al. [34] implemented a dynamic simulation methodology for PV modules’ service lifetime using temperature dependent diffusion and permeation coefficients of polymer films cross-correlated with with real-world time series climatic data. Combined statistical and analytical modeling were performed to predict operation cell temperature using principle component analysis and moisture content on PV modules using finite element models and climate data [35, 36]. This approach was also applied to simulate moisture ingress into PV modules [37]. Whitfield et al. [38] defines a multi-stress condition model in order to predict module failure due to metalization corrosion under the standard damp heat testing. Pickett [39] suggests well-characterized, time-dependent information about in-service conditions, environmental factors on degradation rates, and degradation rate data under accelerated exposure conditions are essential for realistic service lifetime prediction modeling in order to encompass the kinetic effects that control mechanisms such as hydrolysis.

In this work, predictive modeling of PET degradation under four different accelerated weathering exposures was assessed via fixed-effects and mixed-effects regression modeling approaches. Multi-level models for degradation studies with multiple types of materials and exposure conditions were developed to model real-world scenarios under various uncontrolled stressors. Predictive R^2^ calculations for true prediction errors of the models were demonstrated using the leave-one-out cross validation method. Targeted studies supported by these models can lead to predictive models for in-use condition of PV systems and materials.

## Experimental and statistical methods

### Materials

The three PET grades studied include unstabilized (Dupont-Teijin Melinex 454), UV stabilized (Dupont-Teijin Tetoron HB3), and hydrolytically stabilized (Mitsubishi 8LH1) PET. They are referred to as Unstab, UVStab and HydStab, respectively. They are clear films with thicknesses of 75, 50, and 125 *μ*m, respectively. Nuclear Magnetic Resonance (NMR) analysis showed the UV stabilized grade has 1.5 wt. % UV stabilizer with a chemical formula of *C*_22_ *H*_12_ *N*_2_ *O*_4_ (a benzoxazinone type UV stabilizer commercialized under the name of Cyasorb UV 3638 by CYTEC). Although no additive was found in the hydrolytically stabilized grade, the stabilization was achieved by deactivating reactive carboxyl end groups (CEG) [40]. See S1 Appendix for details.

### Study design and exposures

A lab-based, randomized, longitudinal study design [41] was used where seven PET samples from each grade were randomly assigned to four exposure types. All samples were evaluated step-wise over time every 168 hours (one week) for a total of 1176 hours for seven steps. Each sample was measured at every step and one sample was retained (withdrawn from further exposure) at each time step to create retained sample library for subsequent evaluations. The four laboratory-based accelerated conditions are summarized in Table 1.

**Table 1:** Exposure conditions.

Exposure	Condition
DampHeat	Constant exposure at 85°C and 85% RH
FreezeThaw	Cyclic exposure of 20 hours at 70°C and 85% RH and 30 minutes at −40°C
HotQUV	Constant exposure of UVA light at 1.55 *W*/*m*^2^ at 340 nm at 70°C
CyclicQUV	Cyclic exposure of 8 hours of UVA light at 1.55 *W*/*m*^2^ at 340 nm at 70°C and 4 hours of condensing humidity at 50°C in dark

Q-Lab QUV weathering chambers (Model QUV/Spray with Solar Eye Irradiance Control) were used for the UV light exposures (HotQUV and CyclicQUV). The QUV uses UVA-340 fluorescent lamps (280–400 nm), which closely matches the air mass (AM) 1.5 solar spectrum at the wavelengths between 280 and 360 nm. The HotQUV and CyclicQUV exposures had an irradiance of 1.55 *W*/*m*^2^ at 340 nm at 70°C, comparable to approximately 3 times greater than the intensity of AM 1.5 at 340 nm [42]. The CyclicQUV exposure, per ASTM G154 Cycle 4 [43] standard, is a multi-cyclic multi-stressor exposure of alternating sequences of UV light, heat, and condensing humidity designed to mimic outdoor conditions where materials are exposed to morning dew or rain followed by sunlight.

For the heat and humidity exposures (DampHeat and FreezeThaw), Cincinnati Subzero (Model ZPH8) environmental testing chambers were used, as per the IEC 61215 standard [44]. The temperature was reduced to 70°C for the FreezeThaw exposure to keep PET below the glass transition temperature during temperature cycling. PV modules are required to survive 1000 hours of damp heat and 10 cycles of humidity freeze tests with 1) not more than 5% power degradation, 2) no major visual defects, and 3) no changes to the insulation and wet leakage current.

### Evaluations

Yellowness index (YI) and haze (%) were measured using a HunterLab UltrascanPro colorimeter. YI is a measure of the yellowing of a sample and calculated from the UV-Vis transmission spectrum defined by ASTM E313 standard [45]. Polymer yellowing is observed when light absorption occurs near 420 to 440 nm, which decreases *Y*_*CIE*_ and increases YI as shown in Eq 1 where X (red), Y (green) and Z (blue) represent CIE tristimulus values. Haziness, defined by ASTM D1003-13 standard [46], is apparent cloudiness of a sample caused by scattering of light due to bulk scattering from particles, inhomogeneities or impurities, or surface scattering due to topography and roughness. It is calculated using the diffuse (*T*_*diffuse*_) and total transmission (*T*_*total*_) in the spectral range from 380 to 780 nm as shown in Eq 2. Changes in YI and haze (%) are seen as an early indicator of chemical changes in the polymer due to chromophores or crystallites that are formed during weathering degradation, and precursors to embrittlement and mechanical failure [47]. All samples were evaluated at 168 hrs independent of what exposure cycle the samples were currently in. Samples were measured after they had reached room temperature.

$$\begin{array}{c} YI=\frac{100(1.28X_{CIE}-1.06Z_{CIE})}{Y_{CIE}} \end{array}$$ (1)

$$\begin{array}{c} Haze\left(\%\right)=\frac{T_{diffuse}}{T_{total}}\times 100 \end{array}$$ (2)

### Statistical analysis

Statistical models help quantify the relationships between variables. With a predictor (stressor) and a response, a basic linear model often suffices [48–50]. In longitudinal and multi-level studies with multiple predictors, model selection becomes quite complicated. Models must account for differences between material grades, exposure types, and interactions among these factors. Detailed model selection criteria is in S2 Appendix.

In this study, multi-variable linear regression models (fixed-effects or mixed-effects models depending on the between-sample variation) were applied. In these models, covariates (different levels or groups) are categorical variables structured in a multi-level way each with its own variation. Fixed-effects models are usually implemented when samples with repeated measurements behave similarly in a study (i.e., smaller variance between samples). Mixed-effects models are defined as models that implement both fixed effects parameters and random effects [51]. Random effects are defined as unobserved or unmeasured random variables that are incorporated into models when randomness arises due to factors such as measurement uncertainty, differences between samples’ responses, and the leveling structure of the observed data (i.e., large variance between samples). For random effects, estimated deviation in each individual observational unit’s response are considered to explain the overall variation while keeping the same leveled structure in the data.

The correlation structure specified by the random effects determines the statistical significance of the mixed-effects model [52]. Generally, a linear mixed-effects model can be represented in Eq 3 as follows:

$$\begin{array}{c} y=\beta_{0}+\beta_{1}x_{1}+\beta_{2}x_{2}+\cdots +\beta_{n}x_{n}+b_{1}z_{1}+b_{2}z_{2}+\cdots +b_{n}z_{n}+\epsilon \end{array}$$ (3)

where *y* is the response variable (i.e., a measured outcome: YI or haze (%)), *β*_0_ is the intercept of the regression, *β*_1_ through *β*_*n*_ are the fixed effect coefficients (i.e., parameter estimates), *x*_1_ through *x*_*n*_ are the fixed effect variables (i.e., predictors: material and exposure type), *b*_1_ through *b*_*n*_ are the random effect coefficients, *z*_1_ through *z*_*n*_ are the random effect variables, and *ϵ* is the error term.

Adjusted and multiple R^2^, marginal and conditional R^2^, fitted R^2^, and predictive R^2^ are used to determine the significance of the generated models. The predictive R^2^ is derived from the Allen’s predicted residual error sum of squares (PRESS) statistics [53, 54]. It determines the predictive quality of our models better than the traditional R^2^ which is not a good measure of predictive power. Details for the calculation method are in S3 Appendix.

## Results, analysis, and model selection

### Model of yellowness index

**Fig 1:**
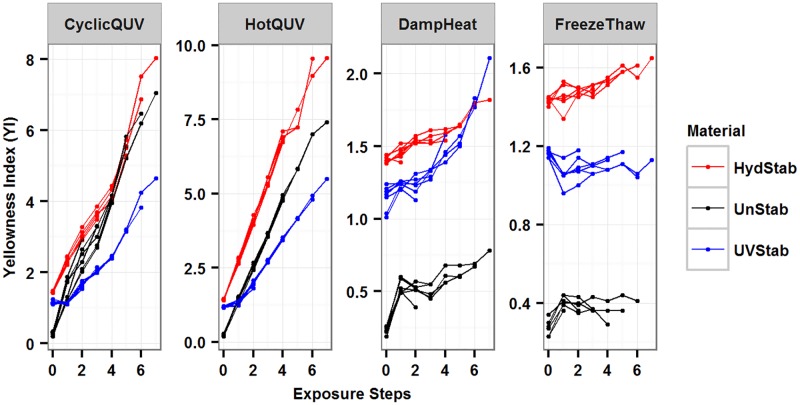
The change in yellowness index (YI) for all material and exposure types as a function of exposure step. HydStab is hydrolytically stabilized PET; UnStab is unstabilized PET; UVStab is UV stabilized PET. Each exposure is plotted on a free scale.

Longitudinal plots of the change in yellowness index (YI) for the three PET grades under the four different types of exposures are shown in Fig 1. YI has different quadratic trends for samples exposed to CyclicQUV and linear trends for samples exposed to HotQUV. The increase in YI is not as pronounced under the DampHeat and FreezeThaw exposures when compared to HotQUV and CyclicQUV. The change in YI in these exposures also suggests different quadratic trends. Therefore, quadratic modeling for all PET grades and exposures and statistical significance of both quadratic and linear terms in the models were assessed.

Exposures that lead to similar trends are analyzed and modeled together to account for the most of the variation. Fig 1 shows a positive steep trend for all materials under the HotQUV and CyclicQUV exposures and a positive gradual trend for materials under the DampHeat and FreezeThaw exposures. Hence samples exposed to HotQUV and CyclicQUV exposures (Model 1) and samples exposed to DampHeat and FreezeThaw exposures (Model 2) are modeled together. The variation in the rate of change in YI across all exposures and materials is small and thus a fixed-effects modeling approach will be used. The models as a function of material and exposure type are shown in Eq 4 for Model 1 and Eq 5 for Model 2. HydStab grade and CyclicQUV exposure in Model 1 and DampHeat exposure in Model 2 are used as reference material and exposures, respectively, and do not appear in the models.

$$\begin{array}{c} \begin{array}{cc} YI & \approx (\beta_{0}+\beta_{01}M_{1}+\beta_{02}M_{2}+\beta_{03}X+\beta_{04}M_{1}X+\beta_{05}M_{2}X)\\ & +(\beta_{1}+\beta_{11}M_{1}+\beta_{12}M_{2}+\beta_{13}X+\beta_{14}M_{1}X+\beta_{15}M_{2}X)t\\ & +\left(\beta_{2}+\beta_{21}M_{1}+\beta_{22}M_{2}+\beta_{23}X+\beta_{24}M_{1}X+\beta_{25}M_{2}X\right)t^{2}\\ & +\left(\beta_{3}+\beta_{31}M_{1}+\beta_{32}M_{2}+\beta_{33}X+\beta_{34}M_{1}X+\beta_{35}M_{2}X\right)t^{3} \end{array} \end{array}$$ (4)

$$\begin{array}{c} \begin{array}{cc} YI & \approx (\beta_{0}+\beta_{01}M_{1}+\beta_{02}M_{2}+\beta_{03}X+\beta_{04}M_{1}X+\beta_{05}M_{2}X)\\ & +(\beta_{1}+\beta_{11}M_{1}+\beta_{12}M_{2}+\beta_{13}X)t\\ & +\left(\beta_{2}+\beta_{21}M_{1}+\beta_{22}M_{2}+\beta_{23}X\right)t^{2}\\ & +\left(\beta_{3}+\beta_{31}M_{1}+\beta_{32}M_{2}+\beta_{33}X\right)t^{3} \end{array} \end{array}$$ (5)

In these model equations *β*’s are parameter estimates, *t* is exposure step, and *M*_1_, *M*_2_, and *X* are as follows:

$$\begin{array}{c} M_{1}=\left\{\begin{array}{cc} 1 & \;\text{if}\;\text{Material}=\text{UnStab}\\ 0 & \;\text{otherwise} \end{array}\right.M_{2}=\left\{\begin{array}{cc} 1 & \;\text{if}\;\text{Material}=\text{UVStab}\\ 0 & \;\text{otherwise} \end{array}\right.X=\left\{\begin{array}{cc} 1 & \;\text{if}\;\text{Exposure}=\text{HotQUV}\;\text{or}\;\text{FreezeThaw}\\ 0 & \;\text{otherwise} \end{array}\right. \end{array}$$

**Fig 2:**
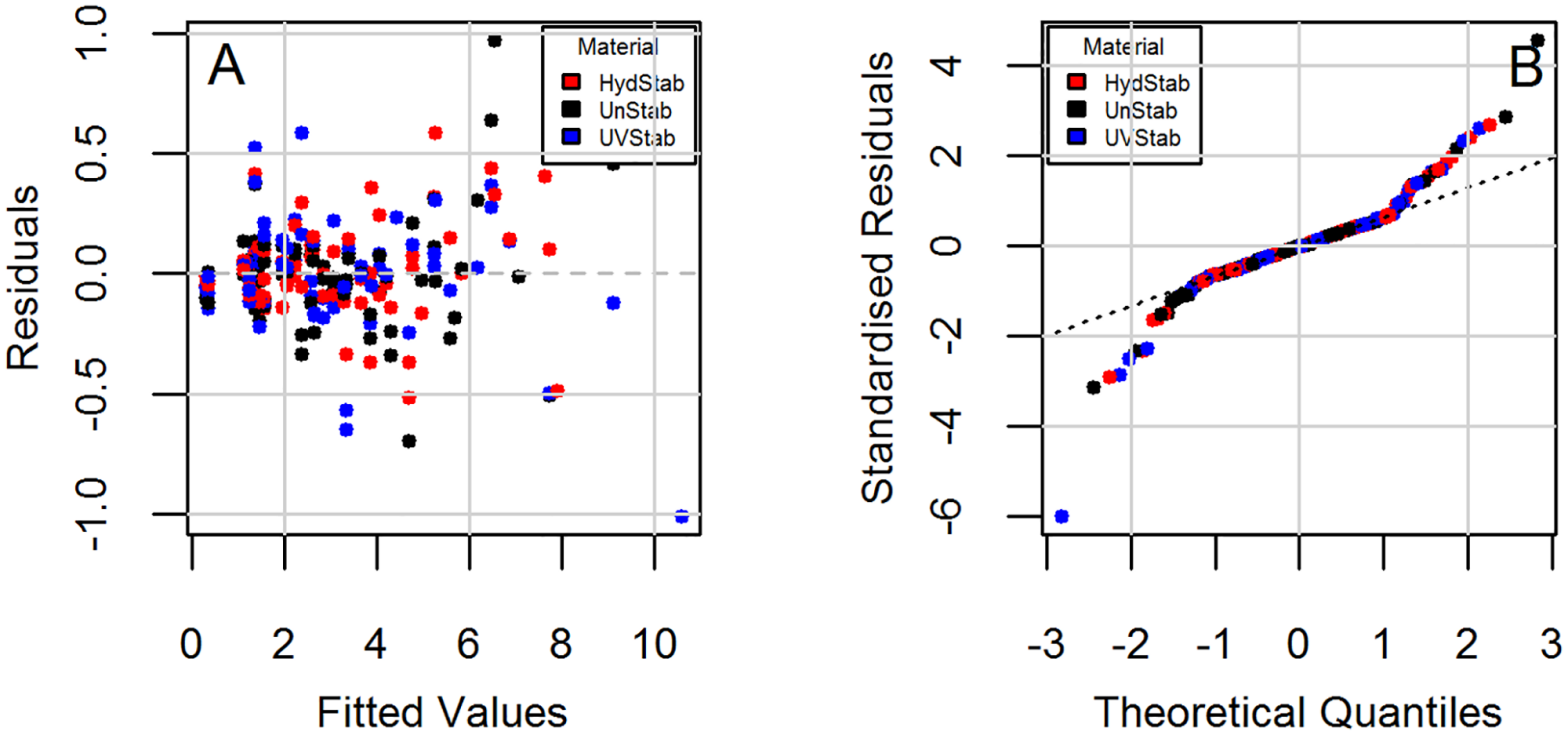
Diagnostic plots for the change in yellowness index (YI) under the HotQUV and CyclicQUV exposures (Model 1). (A) Residuals vs. fitted and (B) Normal Q-Q.

**Fig 3:**
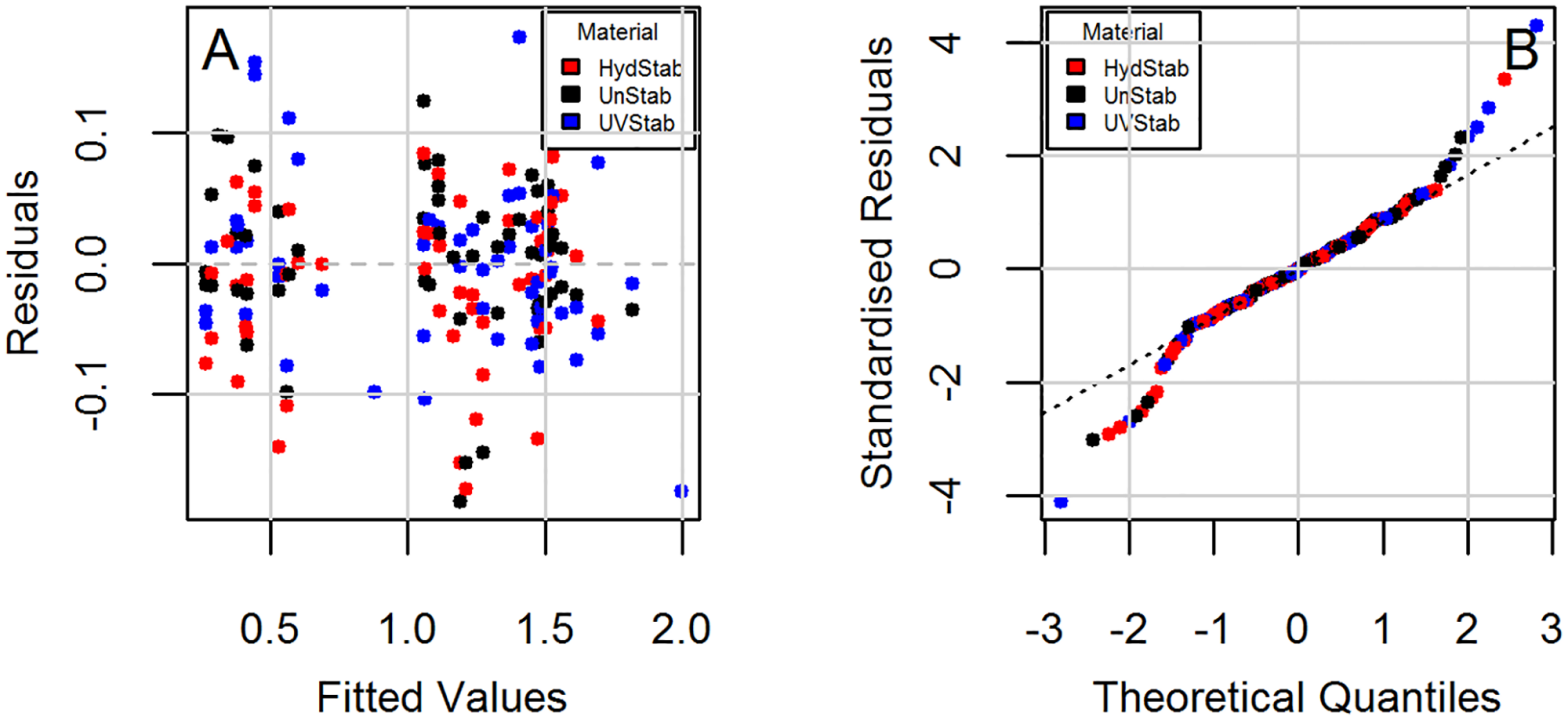
Diagnostic plots for the change in yellowness index (YI) under the DampHeat and FreezeThaw exposures (Model 2). (A) Residuals vs. fitted and (B) Normal Q-Q.

Parameter estimates were then obtained through a step-wise selection procedure (Table 2). It is seen that models do not have interactions between material and exposure in the quadratic and cubic terms. Diagnostic plots to check the regression assumption are shown in Fig 2 for Model 1 and Fig 3 for Model 2. There is no obvious trend in the distribution of points and the residuals are randomly distributed around the horizontal line indicating a mean residual error of close to zero (Figs 2A and 3A). Normal Q-Q plots (Figs 2B and 3B) suggest that the residual errors are normally distributed. These diagnostic plots show that the models follow the experimental data reasonably well.

**Table 2:** Parameter estimates for Model 1 and Model 2.

	*β*_0_	*β*_01_	*β*_02_	*β*_03_	*β*_04_	*β*_05_	*β*_1_	*β*_11_	*β*_12_	*β*_13_	*β*_14_	*β*_15_
Model 1	**1.3563**	−**1.0122**	−**0.2505**	**0.2014**	−**0.2544**	−0.1425	**0.9061**	0.1142	−**0.9212**	**0.3933**	−**0.2679**	−**0.2221**
Model 2	**1.3673**	−**1.1014**	−**0.1755**	**0.1118**	−**0.0908**	−**0.1925**	**0.1031**	**0.1366**	−0.0560	−**0.1175**		
	*β*_2_	*β*_21_	*β*_22_	*β*_23_	*β*_24_	*β*_25_	*β*_3_	*β*_31_	*β*_32_	*β*_33_	*β*_34_	*β*_35_
Model 1	−0.0411	0.0343	**0.1798**				0.0056	−0.0059	−**0.0155**			
Model 2	−0.0216	−**0.0450**	0.0143	**0.0319**			**0.0028**	**0.0036**	−0.0005	−**0.0039**		

The parameter estimates in bold font are found to be significantly different from zero at a 0.05 significance level.

**Fig 4:**
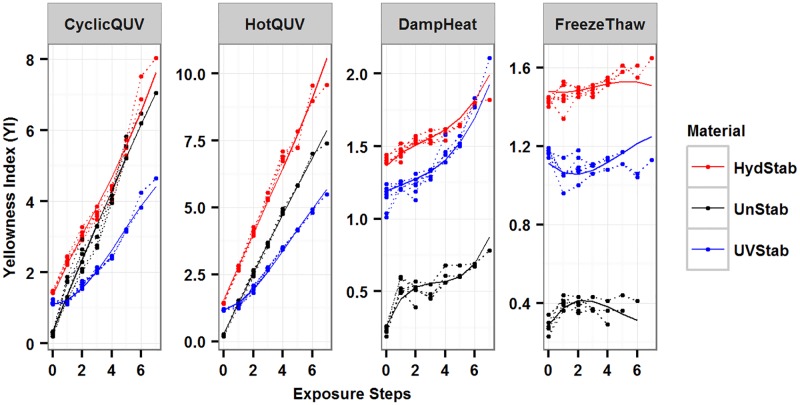
Plot of the change in yellowness index (YI) under all exposures and the generated models to show model fitting. Dashed lines represent the measured data and solid lines represent the models.

The models were overlaid on the observed values for all exposures in Fig 4. Both models explained 98% of the variation in the data from adjusted R^2^ values. The predictive R^2^ value was 0.97 for Model 1 indicating a near perfect prediction power and a true prediction error close to zero. The predictive R^2^ value for Model 2 was 0.46 indicating a relatively larger true prediction error due to possible model over-fitting. The final models’ parameter estimates are shown in Table 2 and details are in S4 Appendix for Model 1 and S5 Appendix for Model 2.

### Model of haze

**Fig 5:**
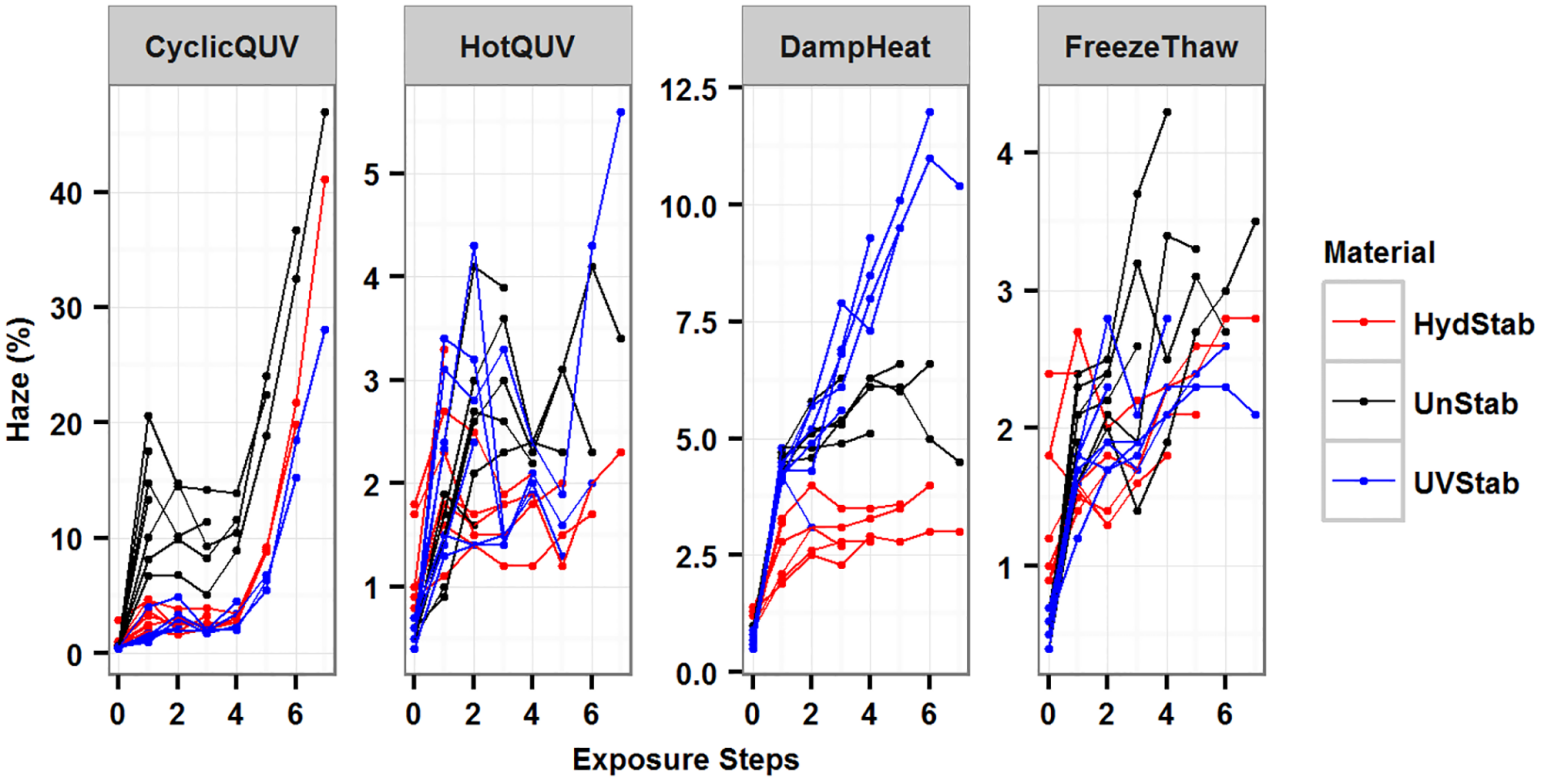
The change in haze (%) for all material and exposure types as a function of exposure step. HydStab is hydrolytically stabilized PET; UnStab is unstabilized PET; UVStab is UV stabilized PET. Each exposure is plotted on a free scale.

Longitudinal plots for the change in haze (%) for the three PET grades under the four different types of exposures are shown in Fig 5. The haze formation has a cubic trend over time for the CyclicQUV exposure while the other exposures show a quadratic trend. Because of measurement sensitivity and variation in haze (%) data between samples, the haze formation depends on individual samples. To account for the behavior of individual samples, a linear mixed-effect model that includes random effects was used.

The haze formation under the CyclicQUV exposure (Model 3, Eq 6) with the combination of high level of humidity content and UV light irradiance is markedly higher than the other three exposure types (Model 4, Eq 7). The HydStab grade and DampHeat exposure are the reference material and exposure, respectively, and do not appear in Model 4.

$$\begin{array}{c} \begin{array}{cc} Haze_{ijkl} & \approx \left(\beta_{0}+\beta_{01}M_{1}+\beta_{02}M_{2}\right)+\left(\beta_{1}+\beta_{11}M_{1}+\beta_{12}M_{2}+b_{1i}\right)t_{ijkl}\\ & +\left(\beta_{2}+\beta_{21}M_{1}+\beta_{22}M_{2}+b_{2i}\right)t_{ijkl}^{2}+\left(\beta_{3}\right)t_{ijkl}^{3}+\epsilon_{ijkl} \end{array} \end{array}$$ (6)

$$\begin{array}{c} \begin{array}{cc} Haze_{ijkl} & \approx (\beta_{0}+\beta_{01}M_{1}+\beta_{02}M_{2}+\beta_{03}X_{1}+\beta_{03}X_{2}+\beta_{04}M_{1}X_{1}+\beta_{05}M_{1}X_{2}\\ & +\beta_{06}M_{2}X_{1}+\beta_{07}M_{2}X_{2})+\left(\beta_{1}+b_{1i}\right)t_{ijkl}+\left(\beta_{2}+b_{2i}\right)t_{ijkl}^{2}+\left(\beta_{3}\right)t_{ijkl}^{3}+\epsilon_{ijkl} \end{array} \end{array}$$ (7)

In these model equations, *β*’s are parameter estimates, *t* is exposure step, *b*_1*i*_ and *b*_2*i*_ are random effects from each sample, *i*(1 ⋯ 7), *j*(1 ⋯ 3), *k*(1 ⋯ 3), and *l*(1 ⋯ 7) represent samples, materials, exposures, and exposure steps, respectively, *ϵ*_*ijkl*_ is the error term, and *M*_1_, *M*_2_, *X*_1_, and *X*_2_ are as follows:

$$\begin{array}{c} M_{1}=\left\{\begin{array}{cc} 1 & \;\text{if}\;\text{Material}=\text{UnStab}\\ 0 & \;\text{otherwise} \end{array}\right.M_{2}=\left\{\begin{array}{cc} 1 & \;\text{if}\;\text{Material}=\text{UVStab}\\ 0 & \;\text{otherwise} \end{array}\right.X_{1}=\left\{\begin{array}{cc} 1 & \;\text{if}\;\text{Exposure}=\text{FreezeThaw}\\ 0 & \;\text{otherwise} \end{array}\right.X_{2}=\left\{\begin{array}{cc} 1 & \;\text{if}\;\text{Exposure}=\text{HotQUV}\\ 0 & \;\text{otherwise} \end{array}\right. \end{array}$$

**Fig 6:**
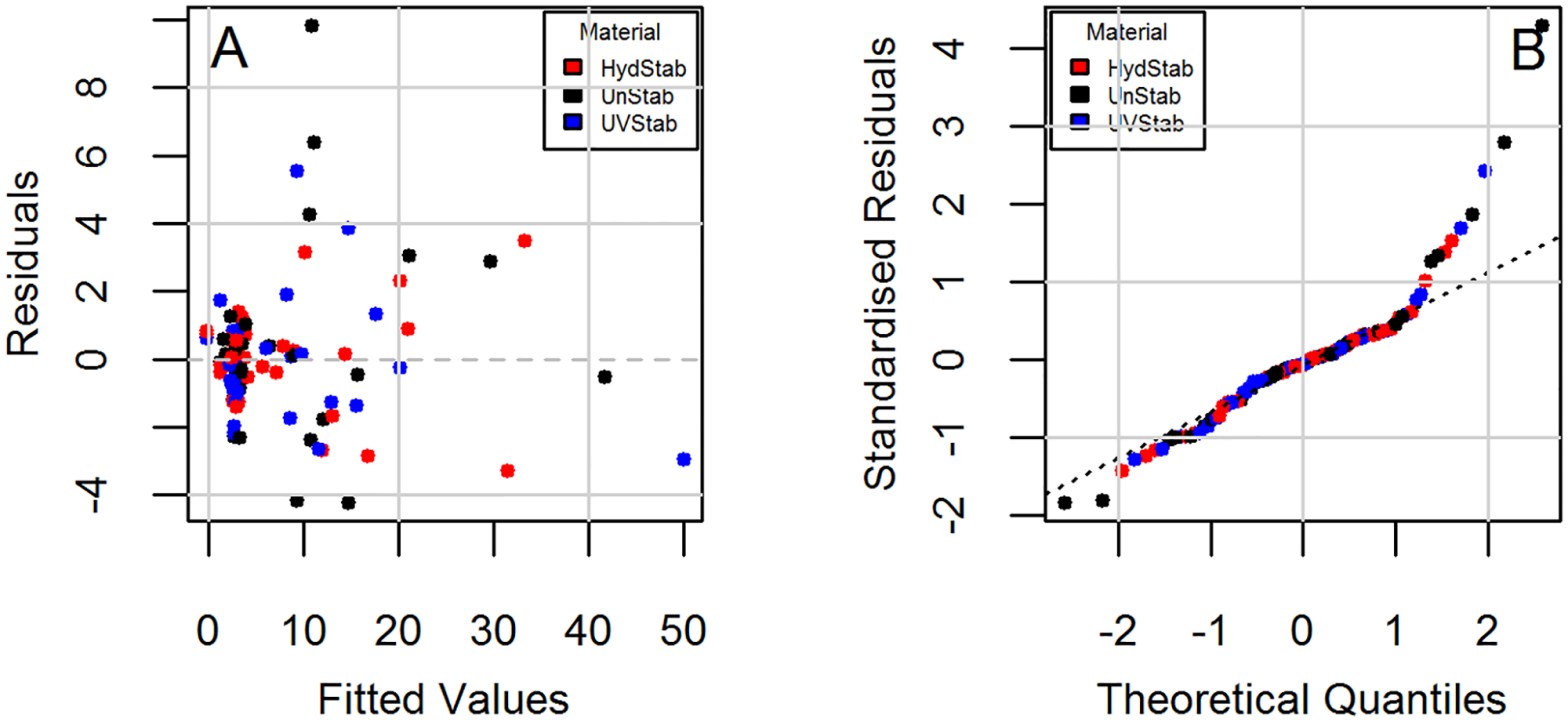
Diagnostic plots for the change in haze (%) under the CyclicQUV exposure (Model 3). (A) Residuals vs. fitted and (B) Normal Q-Q.

**Fig 7:**
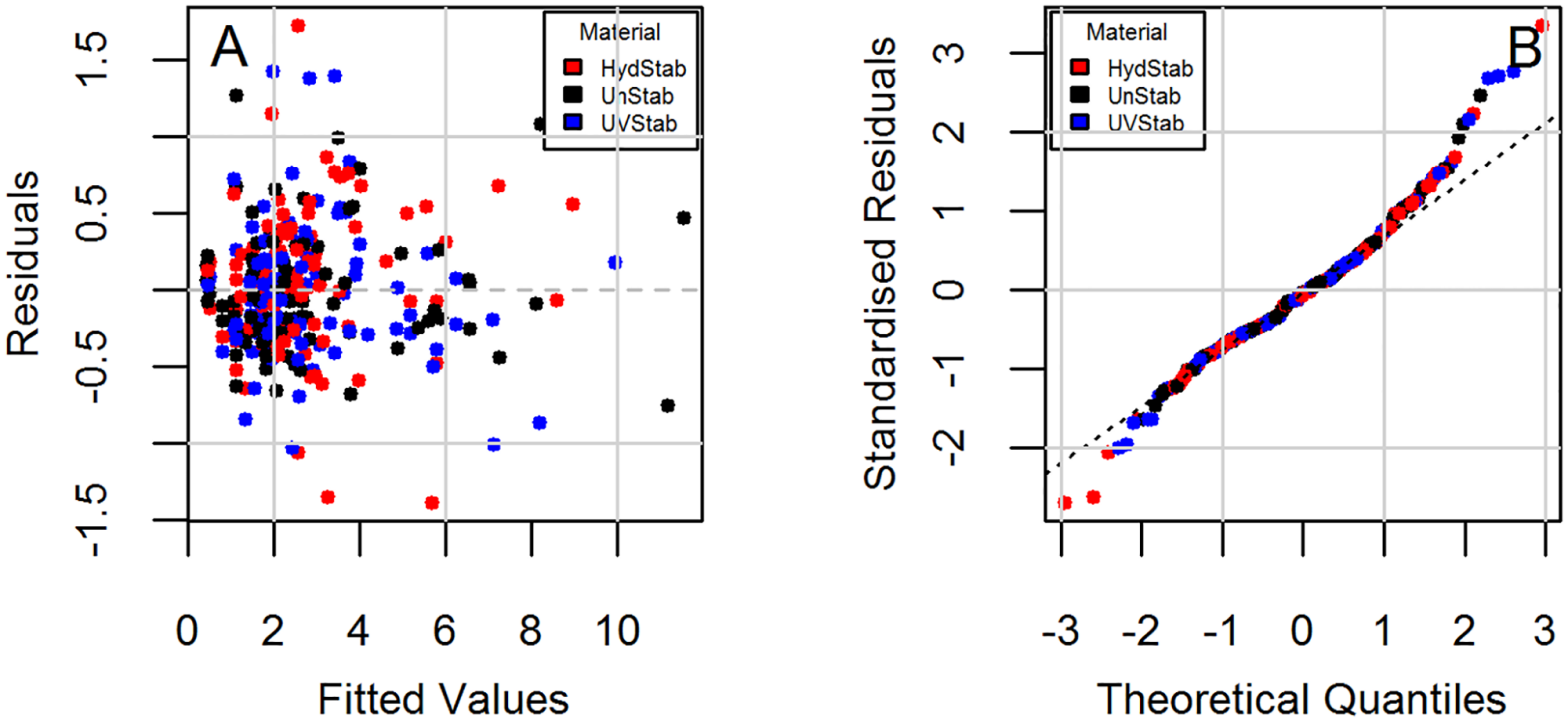
Diagnostic plots for the change in haze (%) under the DampHeat, FreezeThaw, and HotQUV exposures (Model 4). (A) Residuals vs. fitted and (B) Normal Q-Q.

Diagnostic plots checking the regression assumptions are shown in Fig 6 for Model 3 and Fig 7 for Model 4. Model 3 shows different trends for each material type (Fig 6A). The residuals are accumulated around zero residuals error, but only for the fitted values less than 10. The overall trend suggests a possible heteroscedasticity (non-constant variance). This could be due to very high haze (%) values for the last exposure step. Fig 5 shows that a significant change in haze (%) does not begin until the fourth exposure step and it rises markedly afterwards (i.e., onset of haze formation followed by a change point). The reduced sample size from retaining samples at each step may lead to non-constant variance. Also the impact of retained moisture in samples is unknown since samples were not removed during the same cycle in exposures and may have varying concentrations of absorbed water during evaluations. The standardized residuals (Fig 6B) are normally distributed around zero residual errors. Deviation from normality is seen, particularly for the unstabilized grade due to very large increase in its haziness in the very last exposure step. For short-tailed distributions, the result of non-normality does not seem very significant and can be accepted as reasonable [55]. In Model 4, the residual errors (Fig 7A) are distributed randomly and independently from material type as opposed to Model 3. Constant variance and symmetric scattering (Fig 7B) suggests that Model 4 satisfies the regression reasonably well.

**Fig 8:**
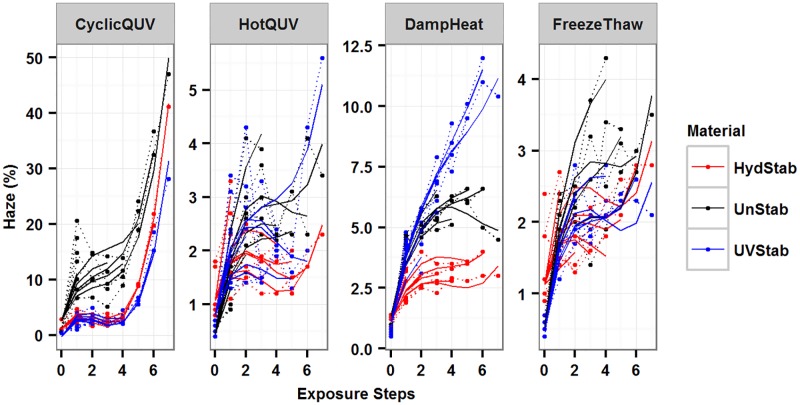
Plot of the change in haze (%) under all exposures and the generated models to show model fitting. Dashed lines represent the measured data and solid lines represent the models.

The models were superimposed on the observed data as shown in Fig 8 for all exposures. The models predict the experimental data very well; however, they do not capture some of the data points as seen in the diagnostic plots. The material and exposure together explained 90% and 31% of the variation in haze formation in Model 3 and Model 4, respectively. Including random effects increased the explained variance to 94% in Model 3 and 93% in Model 4. While fitted R^2^ values were determined to be 0.95 for both models, predictive R^2^ values were calculated to be 0.80 and 0.74 for Model 3 and Model 4, respectively, indicating a relatively small true prediction error for both models. The final model parameter estimates are provided in Table 3. Model summary statistics and the associated random effects can be found in S6 Appendix for Model 3 and S7 Appendix for Model 4.

**Table 3:** Parameter estimates for Model 3 and Model 4.

	*β*_0_	*β*_01_	*β*_02_	*β*_03_	*β*_03_	*β*_04_	*β*_05_	*β*_06_	*β*_07_
Model 3	1.1663	1.4848	−1.4040						
Model 4	**1.1347**	0.2065	−0.0118	−0.0065 (*X*_1_)	−0.0632 (*X*_2_)	−**0.8185**	−**0.8378**	−0.6438	−0.2566
	*β*_1_	*β*_11_	*β*_12_	*β*_2_	*β*_21_	*β*_22_	*β*_3_		
Model 3	**4.1350**	**5.0791**	1.2995	−**2.3959**	−**0.6482**	−0.3133	**0.3749**		
Model 4	**1.8125**			−**0.4429**			**0.0376**		

The parameter estimates in bold font are found to be significantly different from zero at a 0.05 significance level.

## Discussion

### Role of exposures, stressors, and stabilizing additives on degradation modes

The predictive models developed demonstrate important characteristics and differences between discoloration (yellowing) and haze formation in PET films and the categories of stressors present in the different exposure conditions. Yellowing was mostly caused by UV light exposures while haze formation was induced by high humidity exposures. Under the heat and humidity exposures without irradiance, neither degradation mechanisms were found to be pronounced. The effect of stabilization on the UV or hydrolytically stabilized grades was evident from the change points (i.e., onset of yellowing and haze formation). These change points occurred when the degradation mechanisms which led to yellowing or hazing were activated after sufficient damage accumulation. During the induction period, the degradation responses showed little changes as the mechanisms were latent and hindered by the presence of stabilizers. These stabilization techniques did not play a significant role in protecting PET films under the applied exposures. The type of UV stabilizer used was reported to have a poor photo-stability [56]. The rapid exhaustion of the UV stabilizer within a short exposure time caused considerable degradation to the polymer. In the hydrolytically stabilized PET, the diminished carboxyl end group content provided some stabilization. It was slightly more stable than the other two grades under hydrolytic conditions, but this effect was not large [40].

### Yellowing

The fixed-effects modeling was suitable for yellowing due to the small variation in the data across samples. Yellowing is caused by the impact of residual catalysts and the accumulation of degradation byproducts as light absorbing chromophores [57]. Catalysts used in the polymerization give rise to byproducts that accumulate during polymerization and further processing [58]. Degradation byproducts are formed during photolytic or thermo-oxidative degradation in response to applied environmental stressors. Yellowing arises from the strong and broad-band optical absorption of these chromophores in the ultraviolet and visible spectral regions. The chromophores are typically small molecules which form uniformly through the volume of the polymer giving rise to the small observed variation across samples and repeated measurements.

### Haze formation

Hazing is intrinsically a light scattering phenomena and progresses from Rayleigh to Mie scattering as the size of the light scattering entity, which has a contrasting index of refraction from the film, changes its size relative to the wavelength of light [59, 60]. The haze formation in PET films has a number of origins, such as partial crystallinity, i.e., crystallite formation within the bulk material, which can increase due to hydrolysis induced chain scissions leading to increased polymer chain mobility and enabling re-arrangement of amorphous polymer chains into ordered crystalline structures. Volumetric changes from thermal and/or mechanical expansion and contraction in the polymer matrix can cause internal stresses. These internal stresses can produce crazing and cracking in the bulk and/or on the surface, especially as more chain scissions occur due to hydrolysis and temperature cycling. These stressors can also influence the polymer morphology, promoting increased crystallinity. Both reasons for haze formation, crystallites and cracking or crazing, are localized inhomogeneities, not arising from a homogeneous phenomena as is the case for yellowing. Both crystallites and crack formation follow similar nucleation and growth (transformation) kinetics, i.e., time-dependent nuclei formation during which the number of nucleus increase with time, and growth, as governed by well-known Avrami equation [61–63]. This random, localized and distributed formation of the light scattering moieties in a sample leads to measurement sensitivity in the data. The amount of moisture retained inside the samples may be different based on when in the humidity containing exposures samples were removed and if those samples reached equilibrium before evaluation causing a large variation in haziness. Therefore, a mixed-effects model is needed to account for these random effects between samples to accommodate the large variation.

### Prediction errors of models

Predictive R^2^ provides a better statistical measure than adjusted R^2^ in judging model fit and prediction power since it is calculated using the testing data for a model trained on the training data. Comparing adjusted R^2^ to predictive R^2^ also allows one to assess model over-fitting. Apart from the model for yellowing under the DampHeat and FreezeThaw exposures (Model 2), the other models had a predictive power of at least 74%. Adjusted R^2^ values were found to be similar to or greater than the predictive R^2^ values. The low predictive R^2^ indicate that models will not predict new observations as accurately as they fit the existing data, which reduces the accuracy of lifetime prediction.

### Cross-correlation of standards-based tests and real-world performance

In real-world conditions, the stressors are uncontrolled and unpredictable. Modeling approaches based on simple constant stressors and stress levels can not simply be applied for lifetime prediction. Alternatively, under these lab-based, multi-level longitudinal, standardized tests, the fixed- and mixed-effects modeling approaches based on exploratory data analysis have provided accurate prediction for the materials’ behaviors. An advantage of these multi-level models is they account for multiple material types and exposure conditions.

Extending these modeling methods to encompass outdoor exposures will open new opportunities for the development of more reliable lifetime prediction models applicable to in-use conditions. Cross-correlation between lab-based and real-world exposures can elucidate accelerated exposures that more closely mimic outdoor conditions. Well designed, unbiased lab-based and real-world exposures are essential to advance the degradation science of photovoltaic modules.

## Conclusions

The modeling approaches used in this study provide reliable predictions of the changes in yellowing index and haze formation when exposed to constant stressors and stress levels for a given exposure time. Targeted studies supported by these models can lead to predictive lifetime models for in-use condition of PV materials during outdoor deployment where uncontrolled stress conditions make conventional techniques inapplicable. Degradation science studies that combine lab-based and real-world data with predictive models will aid the PV polymer community to improve synthesis and polymerization routes and manufacturing processes and develop materials with increased service life and lifetime performance.

## Supporting information

S1 Appendix Chemical compositions of the PET grades. (PDF)

S2 Appendix Model selection and statistical methods. (PDF)

S3 Appendix Quality of fit and R^2^ values. (PDF)

S4 Appendix Model summary statistics for the change in yellowness index (YI) under HotQUV and CyclicQUV exposures (Model 1). (PDF)

S5 Appendix Model summary statistics for the change in yellowness index (YI) under the DampHeat and FreezeThaw exposures (Model 2). (PDF)

S6 Appendix Model summary statistics for the change in haze (%) under the CyclicQUV exposure (Model 3). (PDF)

S7 Appendix Model summary statistics for the change in haze (%) under the DampHeat, FreezeThaw, and HotQUV exposures (Model 4). (PDF)
