# Supplementary material for: Predictive models of poly(ethylene-terephthalate) film degradation under multi-factor accelerated weathering exposures
Source: PLoS One. 2017 May 12;12(5):e0177614. doi: 10.1371/journal.pone.0177614 (PMC5428936; doi:10.1371/journal.pone.0177614)
Supplement: S1 Appendix — (PDF) [file pone.0177614.s001.pdf]

S1 Appendix. Chemical compositions of the PET grades

The PET grades are commercial products, two were advertised as having stabilization against UV and hydrolytic degradation. Analysis of the three PET grades was performed by Nuclear Magnetic Resonance (NMR) spectroscopy and provided by the 3M Company as part of our collaborative work. The NMR analysis was conducted on a single sample from each grade and should therefore not be taken as a statistically representative sampling of a commercial product production.

The three grades contain standard PET resonances with no co-monomers. The formation of side products and end groups that is known to occur during esterification and polycondensation reactions was also detected. There was no evidence of any additive in the hydrolytically stabilized grade. The stabilization occurred by deactivating reactive polymer chain ends, i.e., carboxyl end groups (CEG), by an end-capping technique, as evidenced by CEG concentrations measured in the initial polymers [1]. For the UV stabilized grade, a compound with a chemical formula of $C_{22}H_{12}N_2O_4$ (a benzoxazinone type UV stabilizer commercialized under the name of Cyasorb UV 3638 by CYTEC) was identified as the UV stabilizer. Pickett [2] reports that this type of UV stabilizer was found to have a poor photo-stability that limits their use in polyesters.

Full chemical compositions of the three grades can be seen in Table S1.1.

Table S1.1. Chemical compositions of the three PET grades.

Grade	TP	EG	DEG	TEG	Veg	MEeg	UV Stabilizer
Hyd. stabilized	68.1	30.4	1.46	0.03	0.01	0.02	-
Unstabilized	68.3	30.8	0.85	0.01	0.02	-	-
UV stabilized	67.4	30.5	0.56	0.01	0.02	0.03	1.5

All values are listed in weight %. TP: terephthalate, EG: ethylene glycol, DEG: diethylene glycol, TEG: triethylene glycol, Veg: vinyl end group, MEeg: methyl ester end group.

References

1. Gok A. Degradation Pathway Models of Poly(ethylene-terephthalate) Under Accelerated Weathering Exposures. Case Western Reserve University; 2016. Available from: `https://etd.ohiolink.edu/pg_10?213511361713081::NO:10:P10_ETD_SUBID:110243`.

2. Pickett JE. Permanence of UV Absorbers in Plastics and Coatings. In: Hamid SH, editor. Handbook of polymer degradation. 2nd ed. No. 21 in Environmental science and pollution control series. New York: Marcel Dekker; 2000. p. 163 – 190.
