# Supplementary material for: Predictive models of poly(ethylene-terephthalate) film degradation under multi-factor accelerated weathering exposures
Source: PLoS One. 2017 May 12;12(5):e0177614. doi: 10.1371/journal.pone.0177614 (PMC5428936; doi:10.1371/journal.pone.0177614)
Supplement: S2 Appendix — (PDF) [file pone.0177614.s002.pdf]

S2 Appendix. Model selection and statistical methods

All statistical computation and modeling were done using R [1] and R Studio [2]. R packages used for analysis and plotting include *lme4* [3], *nlme* [4], *car* [5], *ggplot2* [6], *arm* [7], *RLRSim* [8], and *MuMIn* [9].

The steps applied in model selection for fixed-effects and mixed-effects models are the following:

1. Based on initial inspection of exploratory data analysis (EDA) plots, develop possible models capable of representing the observed trends and their change points. Identify potential outliers using domain knowledge and exclude these from the data during model development.

2. For a given response, exposures showing similar trends are modeled together so as to assure the principle of parsimony in model development.

3. For fixed-effects models;

    (a) Response variable transformation analysis on the initial models are first performed using Box-Cox plots and Tukey's ladder of transformation. These models are comprised of all possible interactions and polynomial terms as required by EDA.

    (b) Linear regression analysis on the models are applied and parameter estimates are obtained.

4. For mixed-effects models;

    (a) Initial models based on EDA plots are hypothesized. Materials and exposures are considered to be fixed effects and sample trends, i.e., linear, quadratic or cubic, are taken as random effects of the models.

    (b) The mixed-effect models are fit using the restricted maximum likelihood (REML) procedure for parameter estimation.

5. Model diagnostic plots are assessed to check for violation of regression assumptions, i.e, normality, heteroscedasticity and linearity. Necessary modifications are made if assumptions are substantially violated.

6. Model fits are performed and determined how, if possible, the models can be simplified without significantly impacting their predictive values so as to identify parsimonious models.

7. Visual inspection by superimposing the applied model on the observed data is performed so as to identify any poorly fit observations.

For the yellowing under DampHeat and FreezeThaw exposures (Model 2), one particular sample's data was identified as outliers since it was showing an opposite trend with the rest of the samples. There were 210 observations in this model's data and the model development was performed on 202 observations after excluding this sample's data.

For the haze formation models, two data points that were having odd behaviors probably due to measurement error or added moisture from removing during the wet cycle were removed from both Model 3's and Model 4's data. There were 105 observations in Model 3 and 315 observations in Model 4 and the model developments were performed on 103 observations in Model 3 and 313 observations in Model 4 after excluding these two data points.

References

1. R: A Language and Environment for Statistical Computing; 2016. Available from: http://www.R-project.org/.
2. RStudio: Integrated Development Environment for R; 2015. Available from: http://www.rstudio.com/.
3. Bates D, Mächler M, Bolker B, Walker S. Fitting Linear Mixed-Effects Models using lme4. Journal of Statistical Software. 2015;67(1):1–48. doi:10.18637/jss.v067.i01.
4. Pinheiro J, Bates D, DebRoy S, Sarkar D, R Core Team. nlme: Linear and Nonlinear Mixed Effects Models; 2015. Available from: http://CRAN.R-project.org/package=nlme.
5. Fox J, Weisberg S. An R companion to applied regression. 2nd ed. Thousand Oaks, CA: SAGE Publications; 2011. Available from: http://socserv.socsci.mcmaster.ca/jfox/Books/Companion.
6. Wickham H. Ggplot2: Elegant Graphics for Data Analysis. New York: Springer; 2009. Available from: http://ggplot2.org.
7. Gelman A, Su YS, Yajima M, Hill J, Pittau MG, Kerman J, et al.. arm: Data Analysis Using Regression and Multilevel/Hierarchical Models; 2016. Available from: https://cran.r-project.org/web/packages/arm/index.html.
8. Scheipl F, Greven S, Küchenhoff H. Size and power of tests for a zero random effect variance or polynomial regression in additive and linear mixed models. Computational Statistics & Data Analysis. 2008;52(7):3283–3299. doi:10.1016/j.csda.2007.10.022.
9. Bartoń K. MuMIn: Multi-Model Inference; 2016. Available from: https://cran.r-project.org/web/packages/MuMIn/index.html.
