# Supplementary material for: Predictive models of poly(ethylene-terephthalate) film degradation under multi-factor accelerated weathering exposures
Source: PLoS One. 2017 May 12;12(5):e0177614. doi: 10.1371/journal.pone.0177614 (PMC5428936; doi:10.1371/journal.pone.0177614)
Supplement: S3 Appendix — (PDF) [file pone.0177614.s003.pdf]

S3 Appendix. Quality of fit and R^2 values

For predictions that use a fixed-effects modeling approach, there are three different R^2 values reported in the model summaries: multiple, adjusted, and predictive. Multiple R^2 is the R^2 value of the multiple regression model and calculated by $1 - SS_{res}/SS_{tot}$, where SS_{res} is the sum of square of residuals, which is the summation of the difference between the predicted value by the model and the observed value, and SS_{tot} is the total sum of squares, which is the summation of the squares of differences between the actual value and the mean value. The difference between multiple and adjusted R^2 comes from the number of variables (k) in the prediction and the number of observations (N) in these variables such that R^2 value is adjusted to account for $(N-1)/(N-k-1)$. In other words, multiple R^2 will improve when a variable is added to the prediction regardless of its significance to the model, but adjusted R^2 will account for model complexity and reflect the corrected measurement error, i.e., it may go up or go down depending on the variance explained by the new variable.

For predictions that use a mixed-effects modeling approach, there are four different R^2 values reported in the model summaries: marginal, conditional, fitted, and predictive.

Marginal and conditional are R^2 values for generalized mixed-effects models calculated using the *r.squaredGLMM* function of the *MuMIn* [1] package that implements a method developed by Nakagawa and Schielzeth [2]. Marginal R^2 provides the variance explained only by fixed effects and conditional R^2 provides the variance explained by the entire model, i.e., both fixed effects and random effects. Fitted R^2 is analogous to adjusted R^2 generalized for measuring explained variation in linear mixed-effects models [3].

The predictive R^2 values assess how well the selected models predict new observations by applying the leave-one-out cross-validation procedure [4], which is based on a re-sampling method. The predictive R^2 is derived from the Allen's predicted residual error sum of squares (PRESS) statistics [5, 6] and used to determine the predictive quality of our models quantitatively since the traditional R^2 is not a good measure of predictive power.

Cross-validation is obtained by dividing the data set into n number of subsets [7]. In each iteration, $n-1$ number of subsets are combined together to train the model and the nth subset which is left out is used to test the same model for estimating the true prediction error, enabling the evaluation of variation in the true error for an unbiased and accurate estimation. The train and test data sets use every data point, but not at the same time. For our study, for the number of data subsets, the extreme case is utilized: leave-one-out, which involves removing one data point (observation) iteratively from the data (i.e., n, the number of subset is equal to the number of observations (N)) and estimating the regression model's true error.

For the calculation of the predictive R^2, let y_{ij} be the observed value for the ith sample at time j, $\overline{y_i}$ is the mean value for the ith sample, N is the number of samples, and $\hat{\mu}^{(-i)}(t_j)$ is the predicted value for the ith sample at time j by the model that is fit using all samples excluding the ith sample. The PRESS value or PSE (predicted squared error) is first computed according to Eq S3.1 as follows.

$$\frac{1}{N} PRESS = PSE = \frac{1}{N} \sum_{i,j} (y_{ij} - \hat{\mu}^{(-i)}(t_j))^2 \tag{S3.1}$$

The total sum of squares is then calculated by Eq S3.2. In PRESS statistics, global average is used for the total sum of squares calculation. However; because each sample in our study has multiple measurements due to step-wise exposures, instead of global average, moving average is calculated for each sample and then the resulting deviations are summed together for the total sum of squares. This results in more precise

predictive R^2 than that calculated with global average. It is to be noted that predictive R^2 values calculated using moving average were reported in the main manuscript, but values calculated by both methods were included here in model summary statistic tables for comparison purposes.

$$SS_{tot} = \sum_{i,j}(y_{ij} - \overline{y_i})^2 \tag{S3.2}$$

And the predictive R^2 can be obtained by Eq S3.3 as follows.

$$\text{Predictive } R^2 = 1 - \frac{PRESS}{SS_{tot}} = 1 - \frac{PSE}{\frac{1}{N}SS_{tot}} \tag{S3.3}$$

Predictive R^2 provides a more accurate measure of model prediction error owing to its re-sampling nature [8]. Other common techniques used to assess this prediction error, such as the adjusted R^2, can sometimes be misleading in the case of over-fitting, that is, where a high value of R^2 (or a low level prediction error) can be achieved with the applied model on the train data; however, applying the very same model to a new test data might result in a poorer prediction [9].

References

1. Bartoń K. MuMIn: Multi-Model Inference; 2016. Available from: `https://cran.r-project.org/web/packages/MuMIn/index.html`.

2. Nakagawa S, Schielzeth H. A general and simple method for obtaining R-squared from generalized linear mixed-effects models. Methods in Ecology and Evolution. 2013;4(2):133–142. doi:10.1111/j.2041-210x.2012.00261.x.

3. Xu R. Measuring explained variation in linear mixed effects models. Statistics in Medicine. 2003;22(22):3527–3541. doi:10.1002/sim.1572.

4. Cawley GC, Talbot NLC. Efficient leave-one-out cross-validation of kernel fisher discriminant classifiers. Pattern Recognition. 2003;36(11):2585–2592. doi:10.1016/S0031-3203(03)00136-5.

5. Allen DM. Mean Square Error of Prediction as a Criterion for Selecting Variables. Technometrics. 1971;13(3):469–475. doi:10.2307/1267161.

6. Allen DM. The Relationship Between Variable Selection and Data Augmentation and a Method for Prediction. Technometrics. 1974;16:125–127.

7. James G, Witten D, Hastie T, Tibshirani R. An Introduction to Statistical Learning: with Applications in R. 1st ed. Springer Texts in Statistics. New York: Springer; 2013. Available from: `http://www-bcf.usc.edu/~gareth/ISL/index.html`.

8. Frost J. Multiple Regression Analysis: Use Adjusted R-Squared and Predicted R-Squared to Include the Correct Number of Variables; 2013. Available from: `http://blog.minitab.com/blog/adventures-in-statistics/multiple-regession-analysis-use-adjusted-r-squared-and-predicted-r-square`

9. Hopper T. Can We do Better than R-squared?; 2014. Available from: `https://www.r-bloggers.com/can-we-do-better-than-r-squared/`.
