# Supplementary material for: Predictive models of poly(ethylene-terephthalate) film degradation under multi-factor accelerated weathering exposures
Source: PLoS One. 2017 May 12;12(5):e0177614. doi: 10.1371/journal.pone.0177614 (PMC5428936; doi:10.1371/journal.pone.0177614)
Supplement: S4 Appendix — (PDF) [file pone.0177614.s004.pdf]

S4 Appendix. Model summary statistics for the change in yellowness index (YI) under the HotQUV and CyclicQUV exposures (Model 1)

Eq S4.1 is the fitted expression using R's *lm* function for fixed-effects modeling and Eq S4.2 is the model expression with parameter estimates. Table S4.1, Table S4.2, and Table S4.3 provide residuals of the fitted model, model summary statistics, and coefficients of the parameter estimates of the fitted model, respectively, for the change in yellowness index (YI) under the HotQUV and CyclicQUV exposures.

$$\begin{aligned} YI &\sim (Step \times Material \times Exposure) \\ &+ (I(Step^2) \times Material) + (I(Step^3) \times Material) \end{aligned} \tag{S4.1}$$

Table S4.1. Residuals of the fitted model for the change in yellowness index (YI) under the HotQUV and CyclicQUV exposures.

Minimum	1st Quantile	Median	3rd Quantile	Maximum
-1.00930	-0.10170	-0.00189	0.09361	0.96893

Table S4.2. Model summary statistics for the change in yellowness index (YI) under the HotQUV and CyclicQUV exposures.

**Residual standard error**	0.2315 on 192 degrees of freedom
**Multiple R^2**	0.9877
**Adjusted R^2**	0.9866
**Predictive R^2 (moving)**	0.9709
**Predictive R^2 (global)**	0.9810
**F-statistic**	906.5 on 17 and 192 degrees of freedom
**p-value**	$< 2.2e-16$

Table S4.3. Coefficients of parameter estimates for the change in yellowness index (YI) under the HotQUV and CyclicQUV exposures.

		Estimate	Std. Error	t value	Pr(>|t|)	
**(Intercept)**	β_0	1.356386	0.073730	18.397	< 2e-16	***
**MaterialUnStab**	β_{01}	-1.012225	0.104270	-9.708	< 2e-16	***
**MaterialUVStab**	β_{02}	-0.250507	0.104270	-2.402	0.017236	*
**ExpHotQUV**	β_{03}	0.201429	0.087501	2.302	0.022407	*
**MaterialUnStab:ExpHotQUV**	β_{04}	-0.254464	0.123745	-2.056	0.041101	*
**MaterialUVStab:ExpHotQUV**	β_{05}	-0.142500	0.123745	-1.152	0.250935	
**Step**	β_1	0.906163	0.091868	9.864	< 2e-16	***
**Step:MaterialUnStab**	β_{11}	0.114249	0.129920	0.879	0.380296	
**Step:MaterialUVStab**	β_{12}	-0.921249	0.129920	-7.091	2.49e-11	***
**Step:ExpHotQUV**	β_{13}	0.393333	0.028241	13.928	< 2e-16	***
**Step:MaterialUnStab:ExpHotQUV**	β_{14}	-0.267902	0.039939	-6.708	2.15e-10	***
**Step:MaterialUVStab:ExpHotQUV**	β_{15}	-0.222173	0.039939	-5.563	8.82e-08	***
**I($Step^2$)**	β_2	-0.041100	0.035440	-1.160	0.247604	
**MaterialUnStab:I($Step^2$)**	β_{21}	0.034350	0.050119	0.685	0.493940	
**MaterialUVStab:I($Step^2$)**	β_{22}	0.179824	0.050119	3.588	0.000423	***
**I($Step^3$)**	β_3	0.005682	0.003664	1.551	0.122560	
**MaterialUnStab:I($Step^3$)**	β_{31}	-0.005953	0.005181	-1.149	0.252000	
**MaterialUVStab:I($Step^3$)**	β_{32}	-0.015538	0.005181	-2.999	0.003068	**

Significance codes for the parameter estimates: 0 '***', 0.001 '**', 0.01 '*' 0.05 '.', 0.1 ' '

$$
\begin{aligned}
YI \approx\ & (\mathbf{1.3563} - \mathbf{1.0122}M_1 - \mathbf{0.2505}M_2 + \mathbf{0.2014}X - \mathbf{0.2544}M_1X - 0.1425M_2X) \\
& + (\mathbf{0.9061} + 0.1142M_1 - \mathbf{0.9212}M_2 + \mathbf{0.3933}X - \mathbf{0.2679}M_1X - \mathbf{0.2221}M_2X)t \\
& + (-0.0411 + 0.0343M_1 + \mathbf{0.1798}M_2)t^2 \\
& + (0.0056 - 0.0059M_1 - \mathbf{0.0155}M_2)t^3
\end{aligned}
\tag{S4.2}
$$
