# Supplementary material for: Predictive models of poly(ethylene-terephthalate) film degradation under multi-factor accelerated weathering exposures
Source: PLoS One. 2017 May 12;12(5):e0177614. doi: 10.1371/journal.pone.0177614 (PMC5428936; doi:10.1371/journal.pone.0177614)
Supplement: S5 Appendix — (PDF) [file pone.0177614.s005.pdf]

S5 Appendix. Model summary statistics for the change in yellowness index (YI) under the DampHeat and FreezeThaw exposures (Model 2)

Eq S5.1 is the fitted expression using R's *lm* function for fixed-effects modeling and Eq S5.2 is the model expression with parameter estimates. Table S5.1, Table S5.2, and Table S5.3 provide residuals of the fitted model, model summary statistics, and coefficients of the parameter estimates of the fitted model, respectively, for the change in yellowness index (YI) under the DampHeat and FreezeThaw exposures.

$$\begin{aligned} YI \sim & (Step \times Material) + (Step \times Exposure) + (I(Step^2) \times Material) \\ & + (I(Step^2) \times Exposure) + (I(Step^3) \times Material) \\ & + (I(Step^3) \times Exposure) + (Exposure \times Material) \end{aligned} \tag{S5.1}$$

Table S5.1. Residuals of the fitted model for the change in yellowness index (YI) under the DampHeat and FreezeThaw exposures.

Minimum	1st Quantile	Median	3rd Quantile	Maximum
-0.181783	-0.034787	0.000063	0.033659	0.182301

Table S5.2. Model summary statistics for the change in yellowness index (YI) under the DampHeat and FreezeThaw exposures.

**Residual standard error**	0.06337 on 184 degrees of freedom
**Multiple R^2**	0.9835
**Adjusted R^2**	0.9820
**Predictive R^2 (moving)**	0.4592
**Predictive R^2 (global)**	0.9702
**F-statistic**	644.4 on 17 and 184 degrees of freedom
**p-value**	< 2.2e-16

$$\begin{aligned} YI \approx & (\mathbf{1.3673} - \mathbf{1.1014}M_1 - \mathbf{0.1755}M_2 + \mathbf{0.1118}X - \mathbf{0.0908}M_1X - \mathbf{0.1925}M_2X) \\ & + (\mathbf{0.1031} + \mathbf{0.1366}M_1 - 0.0560M_2 - \mathbf{0.1175}X)t \\ & + (-0.0216 - \mathbf{0.0450}M_1 + 0.0143M_2 + \mathbf{0.0319}X)t^2 \\ & + (\mathbf{0.0028} + \mathbf{0.0036}M_1 - 0.0005M_2 - \mathbf{0.0039}X)t^3 \end{aligned} \tag{S5.2}$$

Table S5.3. Coefficients of parameter estimates for the change in yellowness index (YI) under the DampHeat and FreezeThaw exposures.

		Estimate	Std. Error	t value	Pr(>|t|)	
**(Intercept)**	β_0	1.367340	0.019822	68.982	< 2e-16	***
**MaterialUnStab**	β_{01}	-1.101406	0.025849	-42.609	< 2e-16	***
**MaterialUVStab**	β_{02}	-0.175557	0.025346	-6.926	7.00e-11	***
**ExpFreezeThaw**	β_{03}	0.111834	0.022720	4.922	1.89e-06	***
**MaterialUnStab:ExpFreezeThaw**	β_{04}	-0.090815	0.022355	-4.062	7.18e-05	***
**MaterialUVStab:ExpFreezeThaw**	β_{05}	-0.192571	0.021421	-8.990	2.98e-16	***
**Step**	β_1	0.103199	0.028900	3.571	0.000454	***
**Step:MaterialUnStab**	β_{11}	0.136627	0.036655	3.727	0.000257	***
**Step:MaterialUVStab**	β_{12}	-0.056071	0.035138	-1.596	0.112257	
**Step:ExpFreezeThaw**	β_{13}	-0.117500	0.029521	-3.980	9.90e-05	***
**I(Step^2)**	β_2	-0.021672	0.011332	-1.912	0.057369	.
**MaterialUnStab:I(Step^2)**	β_{21}	-0.045093	0.014657	-3.077	0.002413	**
**MaterialUVStab:I(Step^2)**	β_{22}	0.014354	0.013718	1.046	0.296765	
**ExpFreezeThaw:I(Step^2)**	β_{23}	0.031959	0.011717	2.727	0.007000	**
**I(Step^3)**	β_3	0.002817	0.001177	2.393	0.017720	*
**MaterialUnStab:I(Step^3)**	β_{31}	0.003611	0.001556	2.322	0.021342	*
**MaterialUVStab:I(Step^3)**	β_{32}	-0.000588	0.001418	-0.415	0.678882	
**ExpFreezeThaw:I(Step^3)**	β_{33}	-0.003911	0.001234	-3.170	0.001785	**

Significance codes for the parameter estimates: 0 '***', 0.001 '**', 0.01 '*' 0.05 '.', 0.1 ' '
