# Supplementary material for: Predictive models of poly(ethylene-terephthalate) film degradation under multi-factor accelerated weathering exposures
Source: PLoS One. 2017 May 12;12(5):e0177614. doi: 10.1371/journal.pone.0177614 (PMC5428936; doi:10.1371/journal.pone.0177614)
Supplement: S6 Appendix — (PDF) [file pone.0177614.s006.pdf]

S6 Appendix. Model summary statistics for the change in haze (%) under the CyclicQUV exposure (Model 3)

Eq S6.1 is the fitted expression using R's *lme* function for mixed-effects modeling and Eq S6.2 is the model expression with parameter estimates. Table S6.1, Table S6.2, Table S6.3, and Table S6.4 provide residuals of the fitted model, model summary statistics, coefficients of the parameter estimates of the fitted model, and the associated random effects due to individual samples, respectively, for the change in haze (%) under the CyclicQUV exposure.

$$\begin{aligned} &\text{Fixed effects : Haze} \sim (\text{Step} \times \text{Material}) + (\text{I}(\text{Step}^2) \times \text{Material}) + \text{I}(\text{Step}^3) \\ &\text{Random effects : Sample} \sim (-1 + \text{Step} + \text{I}(\text{Step}^2)) \end{aligned} \tag{S6.1}$$

Table S6.1. Standardized within-group residuals for the change in haze (%) under the CyclicQUV exposure.

Minimum	1st Quantile	Median	3rd Quantile	Maximum
-1.84065756	-0.47842807	-0.05626121	0.32216072	4.28936316

Table S6.2. Model summary statistics for the change in haze (%) under the CyclicQUV exposure.

**Linear mixed-effects model parameters**	AIC	520.148
	BIC	555.6044
	logLik	-246.074
**Coefficients of generalized mixed-effects model**	Marginal R^2_m	0.9005
	Conditional R^2_c	0.9364
	Fitted R^2_f	0.9502
	Predictive R^2 (moving)	0.8067
	Predictive R^2 (global)	0.8749

Table S6.3. Coefficients of parameter estimates for the change in haze (%) under the CyclicQUV exposure.

		Estimate	Std. Error	DF	t value	p-value
**(Intercept)**	β_0	1.166370	0.7925858	75	1.471601	0.1453
**MaterialUnStab**	β_{01}	1.484816	0.0915512	18	0.360281	0.1905
**MaterialUVStab**	β_{02}	-1.404027	0.0933107	18	-1.284197	0.2154
**Step**	β_1	4.135024	1.1284642	75	3.664294	0.0005
**Step:MaterialUnStab**	β_{11}	5.079136	1.3310445	75	3.815903	0.0003
**Step:MaterialUVStab**	β_{12}	1.299529	1.3248225	75	0.980908	0.3298
**I(Step^2)**	β_2	-2.395967	0.3494089	75	-6.857201	0.0000
**MaterialUnStab:I(Step^2)**	β_{21}	-0.648207	0.2482269	75	-2.611348	0.0109
**MaterialUVStab:I(Step^2)**	β_{22}	-0.313325	0.2465523	75	-1.270826	0.2077
**I(Step^3)**	β_3	0.374944	0.0345852	75	10.841176	0.0000

$$\begin{aligned} Haze_{ijkl} \approx\ & (1.1663 + 1.4848 M_1 - 1.4040 M_2) \\ & + (\mathbf{4.1350} + \mathbf{5.0791} M_1 + 1.2995 M_2 + b_{1i}) t_{ijkl} \\ & + (-\mathbf{2.3959} - \mathbf{0.6482} M_1 - 0.3133 M_2 + b_{2i}) t^2_{ijkl} \\ & + (\mathbf{0.3749}) t^3_{ijkl} + \epsilon_{ijkl} \end{aligned} \tag{S6.2}$$

Table S6.4. Random effects arisen from individual samples for the change in haze (%) under the CyclicQUV exposure.

Samples	Step	I($Step^2$)	Samples	Step	I($Step^2$)
sa19601.22	-0.22312110	0.042253518	sa19603.26	1.94828085	-0.365829294
sa19601.23	-0.13413076	0.024577950	sa19603.27	-1.34873106	0.332002367
sa19601.24	0.23867903	-0.043398515	sa19603.28	-2.54838045	0.432626059
sa19601.25	-0.06231086	0.008362503	sa19604.22	-0.44675886	0.084604878
sa19601.26	-0.48216413	0.092330106	sa19604.23	-0.38338380	0.072438079
sa19601.27	0.71650476	-0.137941910	sa19604.24	-0.42948402	0.081389410
sa19601.28	-0.05345694	0.013816348	sa19604.25	0.81979900	-0.145873570
sa19603.22	1.17336952	-0.222206638	sa19604.26	-0.15979213	0.045997957
sa19603.23	2.37422030	-0.449617535	sa19604.27	0.04918615	-0.013514787
sa19603.24	0.08583301	-0.025737480	sa19604.28	0.55043367	-0.125041967
sa19603.25	-1.68459217	0.298762521			

Samples sa19601, sa19603, and sa19604 are for hydrolytically stabilized, unstabilized, and UV stabilized, respectively, and extensions after periods (.) are for sample replicates.
