# Supplementary material for: Predictive models of poly(ethylene-terephthalate) film degradation under multi-factor accelerated weathering exposures
Source: PLoS One. 2017 May 12;12(5):e0177614. doi: 10.1371/journal.pone.0177614 (PMC5428936; doi:10.1371/journal.pone.0177614)
Supplement: S7 Appendix — (PDF) [file pone.0177614.s007.pdf]

S7 Appendix. Model summary statistics for the change in haze (%) under the DampHeat, FreezeThaw, and HotQUV exposures (Model 4)

Eq S7.1 is the fitted expression using R's *lme* function for mixed-effects modeling and Eq S7.2 is the model expression with parameter estimates. Table S7.1, Table S7.2, Table S7.3, and Table S7.4 provide residuals of the fitted model, model summary statistics, coefficients of the parameter estimates of the fitted model, and the associated random effects due to individual samples, respectively, for the change in haze (%) under the DampHeat, FreezeThaw, and HotQUV exposures.

$$\begin{aligned} &\text{Fixed effects : Haze} \sim \text{Step} + \text{I}(\text{Step}^2) + \text{I}(\text{Step}^3) + (\text{Material} \times \text{Exposure}) \\ &\text{Random effects : Sample} \sim (-1 + \text{Step} + \text{I}(\text{Step}^2)) \end{aligned} \tag{S7.1}$$

Table S7.1. Standardized within-group residuals for the change in haze (%) under the DampHeat, FreezeThaw, and HotQUV exposures.

Minimum	1st Quantile	Median	3rd Quantile	Maximum
-2.69377240	-0.52656940	-0.03260017	0.44209805	3.35057300

Table S7.2. Model summary statistics for the change in haze (%) under the DampHeat, FreezeThaw, and HotQUV exposures.

**Linear mixed-effects model parameters**	AIC	771.3163
	BIC	830.6301
	logLik	-369.6582
**Coefficients of generalized mixed-effects model**	Marginal R^2_m	0.3124
	Conditional R^2_c	0.9330
	Fitted R^2_f	0.9504
	Predictive R^2 (moving)	0.7437
	Predictive R^2 (global)	0.8814

Table S7.3. Coefficients of parameter estimates for the change in haze (%) under the DampHeat, FreezeThaw, and HotQUV exposures.

		Estimate	Std. Error	DF	t value	p-value
**(Intercept)**	β_0	1.1347292	0.1646014	247	6.893801	0.0000
**MaterialUnStab**	β_{01}	0.2065741	0.2284317	54	0.904315	0.3698
**MaterialUVStab**	β_{02}	-0.0118698	0.2283741	54	-0.051975	0.9587
**ExpFreezeThaw**	β_{03}	-0.0065295	0.2296292	54	-0.028435	0.9774
**ExpHotQUV**	β_{03}	-0.0632841	0.2283741	54	-0.277107	0.7828
**MaterialUnStab:ExpFreezeThaw**	β_{04}	-0.8185500	0.3238990	54	-2.527177	0.0145
**MaterialUnStab:ExpHotQUV**	β_{05}	-0.8378123	0.3230104	54	-2.593762	0.0122
**MaterialUVStab:ExpFreezeThaw**	β_{06}	-0.6438805	0.3238584	54	-1.988154	0.0519
**MaterialUVStab:ExpHotQUV**	β_{07}	-0.2566150	0.3229697	54	-0.794548	0.4304
**Step**	β_1	1.8125596	0.1405364	247	12.897439	0.0000
**I(Step^2)**	β_2	-0.4429499	0.0437367	247	-10.127651	0.0000
**I(Step^3)**	β_3	0.0376986	0.0047331	247	7.964921	0.0000

$$\begin{aligned} Haze_{ijkl} \approx (&\mathbf{1.1347} + 0.2065M_1 - 0.0118M_2 - 0.0065X_1 - 0.0632X_2 - \mathbf{0.8185}M_1X_1 \\ &- \mathbf{0.8378}M_1X_2 - 0.6438M_2X_1 - 0.2566M_2X_2) + (\mathbf{1.8125} + b_{1i})t_{ijkl} \\ &+ (-\mathbf{0.4429} + b_{2i})t^2_{ijkl} + (\mathbf{0.0376})t^3_{ijkl} + \epsilon_{ijkl} \end{aligned} \tag{S7.2}$$

Table S7.4. Random effects arisen from individual samples for the change in haze (%) under the DampHeat, FreezeThaw, and HotQUV exposures.

Samples	Step	I($\mathbf{Step^2}$)	Samples	Step	I($\mathbf{Step^2}$)
sa19601.01	0.46953675	-0.040718017	sa19603.12	-0.31783685	0.062766619
sa19601.02	-0.18208398	0.017466198	sa19603.13	0.01952239	-0.021645695
sa19601.03	-0.18837505	0.002049388	sa19603.14	-0.47152862	0.053908584
sa19601.04	-0.38149174	0.036181351	sa19603.15	-0.60480581	0.052448490
sa19601.05	0.20559089	-0.049637690	sa19603.16	-0.38736325	0.028253372
sa19601.06	-0.14109839	0.015141054	sa19603.17	0.59701978	-0.056962687
sa19601.07	-0.34074113	0.015193088	sa19603.18	-0.12347238	-0.003843830
sa19601.08	-0.09670152	0.008385913	sa19603.19	-0.34192974	0.037504921
sa19601.09	-1.02150240	0.084153315	sa19603.20	0.21700824	-0.060207047
sa19601.10	-0.93044983	0.082388895	sa19603.21	-0.01550491	-0.005267559
sa19601.11	-0.92288907	0.094488019	sa19604.01	1.61980310	-0.140468600
sa19601.12	-0.81728734	0.091708785	sa19604.02	0.32848557	-0.039333078
sa19601.13	-0.76648845	0.088201093	sa19604.03	0.98824802	-0.091771343
sa19601.14	-0.44950794	0.025339026	sa19604.04	1.28023462	-0.037933825
sa19601.15	0.58605993	-0.050822855	sa19604.05	1.48956039	-0.093223103
sa19601.16	-1.06422090	0.091279500	sa19604.06	1.20774495	0.002340892
sa19601.17	-0.75878799	0.061084329	sa19604.07	1.39562486	-0.074552110
sa19601.18	-0.45923827	0.019158641	sa19604.08	-0.12827906	0.011124302
sa19601.19	-0.80342651	0.081882563	sa19604.09	-0.15066613	0.011887571
sa19601.20	-0.99327181	0.098310528	sa19604.10	-0.40558449	0.030029471
sa19601.21	-0.72261565	0.052274217	sa19604.11	-0.12826026	0.007088170
sa19603.01	1.03574321	-0.089819188	sa19604.12	-0.49540327	0.061925320
sa19603.02	1.39255439	-0.120761693	sa19604.13	-0.42588941	0.045730203
sa19603.03	1.26979699	-0.118476301	sa19604.14	-0.24918395	-0.001779984
sa19603.04	0.75617859	-0.099713937	sa19604.15	0.08544304	-0.007409582
sa19603.05	0.89036192	-0.078413499	sa19604.16	-0.41826724	0.038282114
sa19603.06	0.83843356	-0.080374754	sa19604.17	-0.81880193	0.070277492
sa19603.07	1.19306106	-0.178069822	sa19604.18	-0.09697980	-0.047117489
sa19603.08	-0.01679303	0.001456284	sa19604.19	-0.74179098	0.067311836
sa19603.09	-0.01880191	-0.001981931	sa19604.20	-0.21606112	-0.021679183
sa19603.10	-0.15279343	0.009432085	sa19604.21	-0.31602813	0.052977244
sa19603.11	0.21619143	0.002553930			

Samples sa19601, sa19603, and sa19604 are for hydrolytically stabilized, unstabilized, and UV stabilized, respectively, and extensions after periods (.) are for sample replicates.
